# Parasite-Induced Changes in the Nervous System of the Shore Crab *Hemigrapsus sanguineus*

**DOI:** 10.3390/ijms27135993

**Published:** 2026-07-03

**Authors:** Elena Kotsyuba, Vyacheslav Dyachuk

**Affiliations:** A.V. Zhirmunsky National Scientific Center of Marine Biology, Far Eastern Branch, Russian Academy of Sciences, Vladivostok 690041, Russia

**Keywords:** trematode metacercaria, *Hemigrapsus sanguineus*, apoptosis, crustaceans

## Abstract

Trematodes are a class of parasitic flatworms with complex life cycles involving multiple hosts. They can influence the physiology and behavior of their intermediate hosts to improve the efficiency of transmission to definitive hosts, including by increasing host mortality. However, the mechanisms of their effect on the crustacean nervous system leading to neuronal dysregulation and its consequences have not been sufficiently elucidated. This study investigated the effects of infection by metacercariae of the trematode *Cercaria fluviocinguli* on the serotonin (5-HT) immunoreactivity, inflammation, and pathological processes in the nervous system of the shore crab *Hemigrapsus sanguineus*. The regions in the brain, the ventral nerve cords (VNC), and the nerves targeted by this parasite were examined by immunohistochemistry and confocal laser scanning microscopy methods. The cysts and the enclosed flukes caused compaction, compression, and distortion of many nerve fibers of the VNC and also impaired conduction in the mixed nerves of the thoracic ganglia controlling locomotion. The infection by *C. fluviocinguli* induced a decrease in 5-HT-like immunoreactivity in neurons and neuropils of the VNC, damage to nerve fibers and neurons of the VNC, inflammatory reactions, and apoptosis. In heavily infected crabs, the increased content of TUNEL-positive cells in the VNC was correlated with granuloma accumulation. Further investigations into host–parasite interaction mechanisms under controlled experimental conditions are needed to clarify the actual effects of encapsulated and non-encapsulated metacercariae on host behavior and their role in transmission to the next host.

## 1. Introduction

Many parasitic trematodes with complex life cycles can influence the physiology and behavior of their intermediate hosts to improve the efficiency of transmission to definitive hosts [1,2,3,4,5], including by increasing host mortality [6,7].

**Figure 1:**
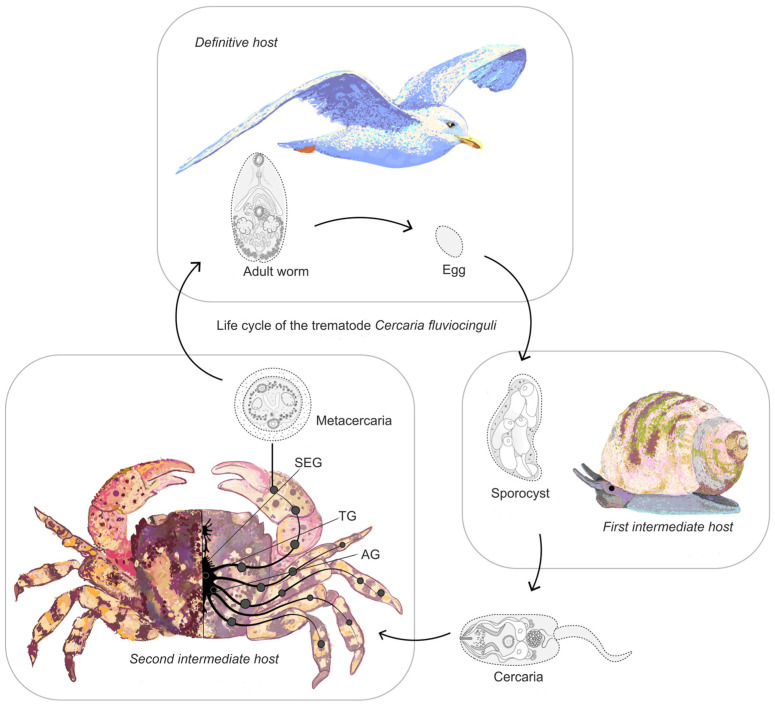
Life cycle of the trematode *Cercaria fluviocinguli.* Letter designations: SEG, subesophageal ganglion; TG, thoracic ganglion; AG, abdominal ganglion.

The genus *Maritrema* Nicoll, 1907 (Digenea, Microphallidae) is characterized by a cosmopolitan distribution. Adult individuals of this genus are found as intestinal parasites in most vertebrate classes, predominantly in aquatic and wetland birds of freshwater, brackish-water, and marine ecosystems worldwide [8,9]. Larvae of *Cercaria fluviocinguli*, belonging to this genus, develop in several mollusk and crustacean species, while adults parasitize the intestines of shorebirds from the genus *Larus*. Their life cycle involves gastropod mollusks as the first intermediate host (*Littorina mandshurica*, *Nucela heuseana,* and *Littorina mandshurica*), in which cercariae develop, and crabs (such as *Hemigrapsus sanguineus*, *H. penicillatus*, and *Cancer amphioetus*) as the second intermediate hosts, in which metacercariae develop (Figure 1). In the second intermediate host, *C. fluviocinguli* metacercariae affect an important part of the central nervous system (CNS): the ventral nerve cord (VNC) and the main nerve roots extending from the thoracic ganglion. Microphallid trematodes are known to use different strategies for infecting their intermediate hosts and can alter the host’s physiology and behavior by modulating neural processes to provide behavioral control that facilitates transmission to the definitive host. However, the mechanisms of their effect on the crustacean nervous system and its consequences have not been sufficiently elucidated.

Parasite-induced alterations in the structure and function of the nervous system that increase the likelihood of transmission to the definitive host have been identified in both vertebrates and invertebrates, including crustaceans [3,10,11,12,13]. Trematodes may employ mechanisms associated with mechanical disruption of the nervous tissue at the site of invasion [14]. In addition, they can affect neuromodulation by changing neurotransmitter balances, which alters the neural circuits and the host’s behavior [10,15].

In recent years, growing evidence has shown that the serotonergic signaling system is involved in host–parasite interactions. Parasite-induced changes in serotonin (5-hydroxytryptamine, or 5-HT) concentrations in the nervous and endocrine systems can cause dysfunction of neuronal processes and alter the behavior of infected hosts [10,11,16,17]. It has been demonstrated that 5-HT concentrations in the brain and/or hemolymph are changed in crustaceans infected by acanthocephalans [18,19,20] and trematodes [21,22], in fish infected by cestodes and trematodes [23,24], and in rodents infected by the nematode *Trichinella* spp. [25].

In crustaceans, as in other invertebrates and in vertebrates, 5-HT is a conserved modulator of behavior [26], playing an important role not only in the nervous and endocrine systems but also in neuroimmune interactions, immune responses, and hematopoiesis [27]. Parasites can influence 5-HT signaling and behavior by hijacking the host immune system to release neuroactive substances or by causing neuroinflammation [10,11,17,28]. In particular, the invasion of cerebral larvae of *Microphallus papillorobustus* causes chronic inflammation in the brain of gammarids, which leads to disruption of 5-HT signaling and changes in the behavior of infected hosts [10,11,16,29,30]. Inflammatory responses depend on a variety of factors such as the species of parasite, its life cycle and biology, induced immune responses, antigenic variability, and immune escape strategy elicited by the parasite [31]. Consequences of cerebral parasitic infections in vertebrates and invertebrates result from a complex interplay between the parasite and the host inflammatory response, which may disrupt brain homeostasis, influence neurotransmission, and lead to significant behavioral changes and, eventually, death [10,11,16,30,32]. For instance, microphallid metacercariae are known to cause tissue damage and activate immune responses that can affect the host fitness [33,34].

In crustaceans, infection can induce oxidative stress resulting in the suppression of antioxidant enzyme activity and, thus, cell damage, DNA damage, and cell death [35]. It can also cause the production of enzymes of protease families capable of initiating the signaling cascades that lead to programmed cell death (apoptosis) [36,37,38]. In both vertebrates and invertebrates, apoptosis is essential for normal tissue homeostasis and also plays a major role in host immune defense by preventing the spread of parasites [39,40,41,42]. Some parasites are able to induce apoptosis to escape the host defense systems [40,42], whereas others can inhibit apoptosis to establish a sustainable interaction with the host [40,43,44]. Previous studies showed that helminths and their products can trigger apoptosis pathways in host immune cells [45,46]. However, to date, the damage that trematodes cause to the crustacean nervous system has been described only in a few reports [29,30]. The shore crab *Hemigrapsus sanguineus* (De Haan, 1835) is one of the crab species most heavily infected by metacercariae of *Cercaria fluviocinguli* (family Microphallidae). Nevertheless, the specific effects of this trematode on such a host remain poorly understood.

In the present study, immunocytochemical experiments, followed by confocal microscopy, were conducted to elucidate the effects of *C. fluviocinguli* metacercariae on the 5-HT-like immunoreactivity and the inflammatory and pathological processes in nerve tissues of shore crabs.

## 2. Results

### 2.1. Distribution of C. fluviocinguli Metacercariae in the CNS of Hemigrapsus sanguineus

**Figure 2:**
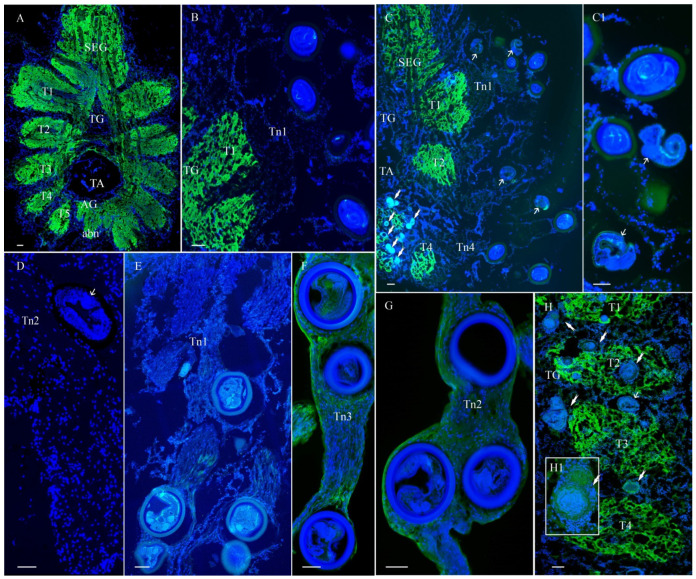
Ventral nerve cord (VNC) and distribution of metacercariae in shore crab, *Hemigrapsus sanguineus*. (**A**) Horizontal section through VNC formed by fusion of subesophageal ganglion (SEG), thoracic ganglion (TG), and abdominal ganglion (AG). (**B**) Localization of metacercariae in the area of segmental nerve (Tn1). (**C**,**C1**) Large aggregation of metacercariae in the area of segmental nerves (Tn1–4). (**C**) Invasion with massive infiltration by hemocytes and granuloma-like formations (long arrows) in TG; unencysted metacercariae (short arrows). (**D**) Metacercaria attached to Tn2 (short arrow). (**E**–**G**) Localization of metacercariae in TG nerves. (**H**) Hemocytic aggregate and granuloma-like formations in TG (long arrows) in a heavily infected crab; metacercariae (short arrow). (**H1**) Granuloma-like formations. Letter designations: SEG, subesophageal ganglion; TA, thoracic artery; T1–T5, neuropils of TG; AG, abdominal ganglion; Tn1–5, segmental nerves; abn, abdominal nerve. Color designations: green, synapsin; blue, DAPI. Scale bars: 100 μm.

Metacercariae of the trematode *C. fluviocinguli*, a stage invasive for crabs, were found in the nervous system of *H. sanguineus.* They were predominantly encysted within the ventral nerve cord (VNC) and nerves of the thoracic region (Figure 1 and Figure 2). However, the brain and the eyestalks in all of the crabs examined were free of metacercariae. In addition, cysts were detected within muscles, especially near the legs, and also in the connective tissue near the VNC.

In *H. sanguineus*, as in other brachyuran crabs, the VNC is represented by a large thoracic ganglion formation consisting of the fused subesophageal (SEG), thoracic (TG), and abdominal (AG) ganglia, from which all peripheral nerve roots extend (Figure 2A). Sections showed that the most common sites of invasion in all groups of infected crabs were the VNC nerves originating from the ganglion in the thoracic region (Figure 2B–G). However, metacercariae in the VNC ganglia were observed in only 33% of heavily infected individuals.

In the infected crabs, the number of metacercariae varied from 1 to 5 to 65. The diameter of encysted metacercariae ranged from 260 to 390 μm. In many sections, encysted *C. fluviocinguli* metacercariae remained in a folded configuration and were surrounded by a thick, two-layered cyst. In addition, histological sections revealed metacercariae with a thin, single-layered cyst wall and unencysted metacercariae that were located in the connective tissue near the nerves and within the VNC nerves (Figure 2C,C1, indicated by arrows). The proportion of unencysted metacercariae in these sections ranged from 3 to 8.3%. In some nerves, several metacercariae were present at a time, arranged either along the nerve fibers and/or transversely, which changed the nerve architecture of the leg (Figure 2E–G). In some nerves or near the nerves, empty cysts were also observed, indicating dead metacercariae (Figure 2G). In the heavily infected crabs, the VNC, mainly in the thoracic region, showed pronounced alterations in the neuropil architecture with numerous empty spaces among nerve fibers, heavy hemocytic infiltration, hemocytic aggregates, melanized foci, and unencysted metacercariae (Figure 2C,H). A characteristic feature of infected crabs was the presence of granulomas in the VNC, whose number increased to 11–13 (per section) in heavily infected crabs. Granuloma-like formations varied in diameter but typically consisted of a large core of degraded remnants of larvae or damaged tissues encapsulated by several layers of flattened hemocytes (Figure 2H,H1).

### 2.2. Effect of C. fluviocinguli Metacercariae on 5-HT-lir

**Figure 3:**
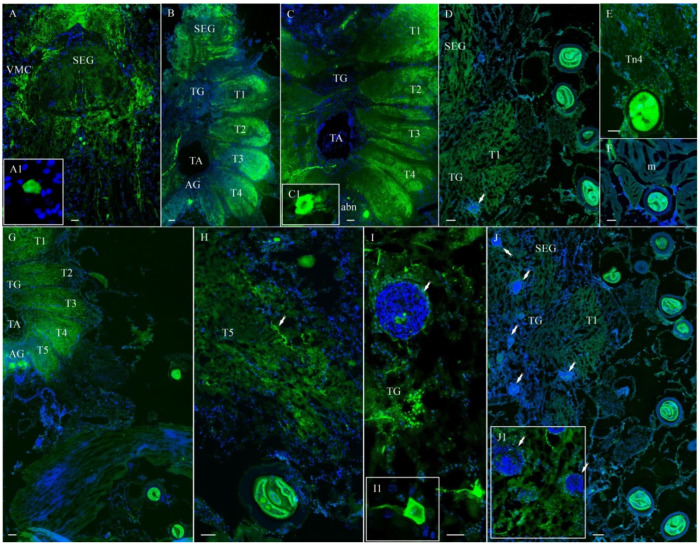
5-HT-lir in crabs, *Hemigrapsus sanguineus*, uninfected (**A**–**C**) and infected (**D**–**I1**) by *Cercaria fluviocinguli*. (**A**) Immunolocalization of 5-HT-lir in the subesophageal ganglion (SEG). (**A1**) 5-HT-lir in a neuron in the ventral nerve cord (VNC). (**B**) Mid-ventral section through VNC showing 5-HT-lir in SEG, TG, and AG. (**C**) 5-HT-lir in thoracic neuropils of TG and in neurons of AG. (**C1**) 5-HT-lir in a neuron in TG. (**D**) Decrease in 5-HT-lir in SEG and TG; high 5-HT-lir in metacercariae; granuloma-like formations (long arrow). (**E**) Localization of metacercariae in nerve. (**F**) Localization of metacercariae in muscle. (**G**) 5-HT-lir in AG neurons. (**H**) 5-HT-lir nerve fibers in TG (long arrow). (**I**) 5-HT-lir in small nerve fibers; small neurons and granuloma-like formation (long arrow) in TG. (**I1**) Small neurons in TG of infected crabs. (**J**) Increase in the number of granuloma-like lesions in TG of heavily infected crabs; granuloma-like formations (long arrows). (**J1**) granuloma-like lesions in TG. Letter designations: SEG, subesophageal ganglion; TG, thoracic ganglion; AG, abdominal ganglion; VMC, ventro-medial clusters; TA, thoracic artery; T1–T5, neuropils of TG; Tn1–5, segmental nerves; abn, abdominal nerve; m, muscle. Color designations: green, 5-HT; blue, DAPI. Scale bars: 100 μm.

Infected and uninfected crabs were compared to determine morphological differences in 5-HT immunoreactivity in the VNC. In the uninfected crabs, thin, 5-HT-like immunoreactive (5-HT-lir) fibers in the VNC formed dense, strongly immunoreactive networks in all neuropils of the SEG, TG, and AG (Figure 3A–C). In the SEG, 5-HT labeled commissural fibers and fibers that extended throughout its length to the TG (Figure 3A,B). In addition, single 5-HT-lir neurons 20–30 μm in diameter were present in both dorsolateral and ventromedial clusters (Figure 3A,A1). In the TG, all neuropils (T1–T5) contained a dense plexus of strongly immunoreactive fibers of 5HT-lir neurites and varicosities (Figure 3B,C). Single, 20–35 μm, 5-HT-lir cells were present in all cell groups in the thoracic segment. In the AG, neuropils were stained weakly for serotonin (Figure 3B,C); however, single small neurons (10–15 μm in diameter) and a pair of giant 5-HT-lir neurons (70–75 μm) were intensely labeled (Figure 3B–C1).

In infected crabs, the intensity of labeling of fibers and neurons in the SEG, TG, and nerves decreased compared to the uninfected individuals (Figure 3D,E). Simultaneously, 5-HT signal of high intensity was detected in metacercariae localized in both nervous and muscle tissues (Figure 3D–I). Among the moderately infected crabs, 5-HT-lir was present only in a pair of giant (70–75 μm), 5-HT-lir AG neurons and in some fibers in the neuropils of the TG in the crabs that had metacercariae localized near the AG (Figure 3G). In the moderately infected crabs that had metacercariae localized in nerves, a decrease in 5-HT labeling intensity was observed in small varicose fibers in all neuropils of the SEG, TG, and AG and leg nerves, but single 5-HT-lir neurons and small nerve fibers were sometimes found in the neuropils of the TG (Figure 3H,I,J1). In the heavily infected crabs, 5-HT-lir in neurons and nerve fibers decreased to complete absence. However, neuropils of the VNC contained numerous hemocytic aggregates and granulomas; in some of them, 5-HT-lir was found (Figure 3D,3I–J1).

### 2.3. Apoptosis-like Activity in Host Tissue

**Figure 4:**
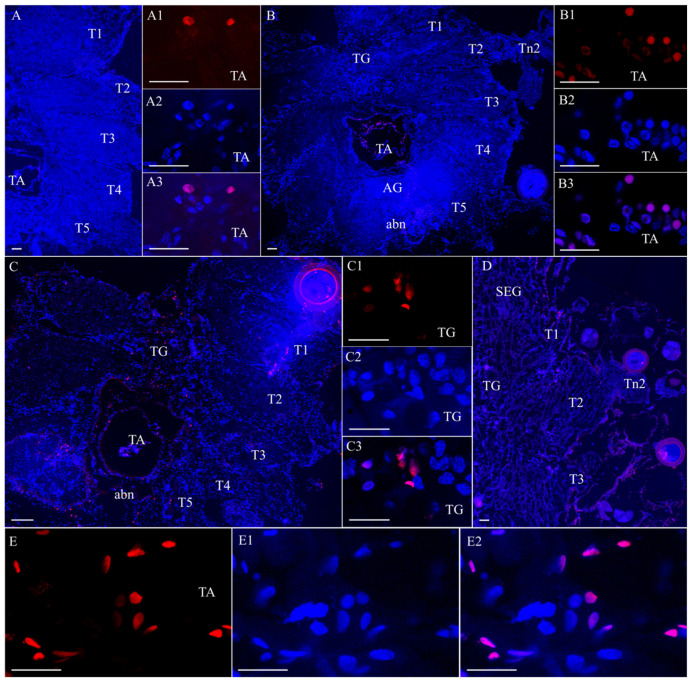
TUNEL assay in ventral nerve cord (VNC) of crabs, *Hemigrapsus sanguineus*, uninfected (**A**–**A3**) and infected (**B**–**E2**) by *Cercaria fluviocinguli*. (**A**–**A3**) Horizontal sections through mid-ventral planes of a part of VNC showing a single TUNEL-positive hemocyte in the thoracic artery of control crabs. (**B**) Mid-dorsal section showing numerous TUNEL-positive hemocytes in the thoracic artery of moderately infected crabs, with localization of metacercariae near VNC. (**C**) Mid-ventral section with a single dying metacercariae localized in VNC neuropil, showing TUNEL-positive cells in VNC of moderately infected crabs; TUNEL-positive neurons (**C1**–**C3**). (**D**) TUNEL-positive cells in VNC of heavily infected crabs. (**E**–**E2**) TUNEL-positive cells in the thoracic artery of heavily infected crabs. Letter designations: SEG, subesophageal ganglion; TG, thoracic ganglion; AG, abdominal ganglion; TA, thoracic artery; T1–T5, neuropils of TG; Tn1–5, segmental nerves; abn, abdominal nerve. Color designations: red, TUNEL staining; blue, DAPI staining. Scale bars: (**A**–**D**), 100 μm; (**A1**–**A3**,**B1**–**B3**,**C1**–**C3**,**E**–**E2**), 25 μm.

Host cells undergoing DNA fragmentation were monitored using the TUNEL assay to clarify the role of parasites in VNC cell death. In the VNC of the uninfected (control) crabs, few TUNEL-positive hemocyte nuclei were detected, primarily in the thoracic artery (Figure 4A–A3). In the moderately infected crabs with metacercariae encysted within nerves, there was an increased number of apoptosis-like cells in the thoracic artery of the VNC compared to the control group (Figure 4A1–A3,B1–B3). Furthermore, in this group, the crabs with single metacercariae in the VNC neuropil had TUNEL-positive neurons and hemocytes in the TG and near metacercariae (Figure 4C–C3). In the heavily infected crabs, the VNC, mainly the TG, contained hemocytic aggregates and granulomas of various shapes and sizes where significant aggregations of TUNEL-positive cells were detected (Figure 4D). In addition, TUNEL-positive cells of different types were found in the thoracic artery (Figure 4E–E2).

**Figure 5:**
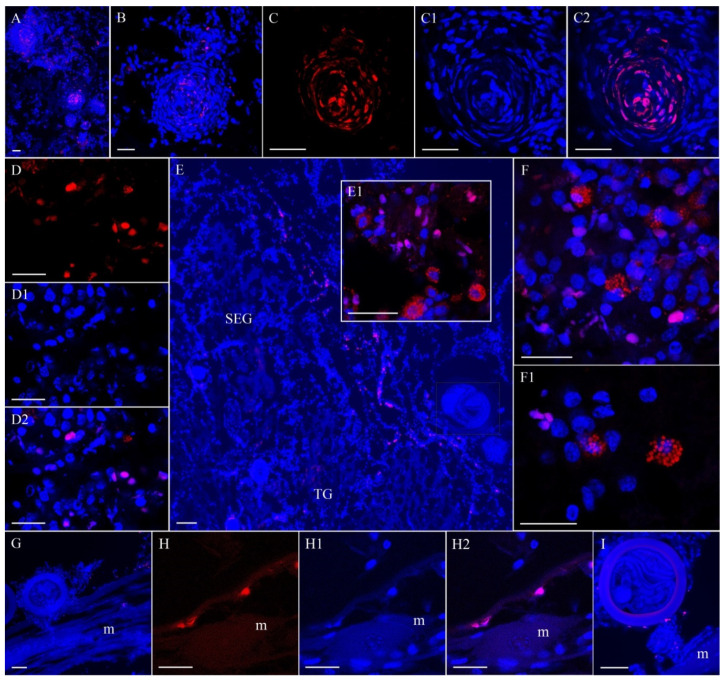
TUNEL assay in ventral nerve cord (VNC) in crabs, *Hemigrapsus sanguineus*, heavily infected by *Cercaria fluviocinguli*. (**A**–**C2**) Hemocytic aggregates and granulomas of different sizes containing TUNEL-positive cells in VNC. (**D**–**D2**) Apoptosis in a cell cluster of TG. (**E**–**F1**) TUNEL-positive cells and an increased number of cells with cytoplasmic TUNEL signal near VNC. (**G**–**H2**) TUNEL-positive cells in muscle tissues. (**I**) TUNEL-positive hemocytes recorded at the host–parasite interface. Letter designations: SEG, subesophageal ganglion; TG, thoracic ganglion; m, muscle. Color designations: red, TUNEL staining; blue, DAPI staining. Scale bars: (**A**,**B**,**E**,**G**,**I**), 100 μm; (**C**–**D2**,**F**,**F1**,**H**–**H2**), 25 μm.

Granulomas were characterized by a central region rich in hemocytes interspersed with numerous TUNEL-positive cells of various sizes and surrounded by multiple layers of elongated, spindle-like cells (Figure 5A–C,C2). TUNEL-positive neurons were found in cell clusters in the TG and AG (Figure 5D–D2). The fluorescence intensity of nuclei in the cell clusters was not homogeneous, suggesting that apoptosis was induced at different times. In the heavily infected crabs, numerous TUNEL-positive granulocytes were also detected, of which most were recorded from the surrounding tissues near the TG and nerves (Figure 5E,E1–F1), and only rare granulocytes were occasionally present near granulomas and some of the metacercariae (Figure 5B). Similar cells were also detected near dying metacercariae in the moderately infected crabs (Figure 4C). Cytoplasmic TUNEL signal was detected in some of the granulocytes (Figure 5E1–F1). A remarkable finding was the TUNEL-positive satellite cells in a leg muscle (Figure 5G,H–H2) that were localized along the surface of muscle fibers (Figure 5H–H2). In addition, we also found TUNEL-positive hemocytes near metacercariae in crab leg muscles (Figure 5I).

**Figure 6:**
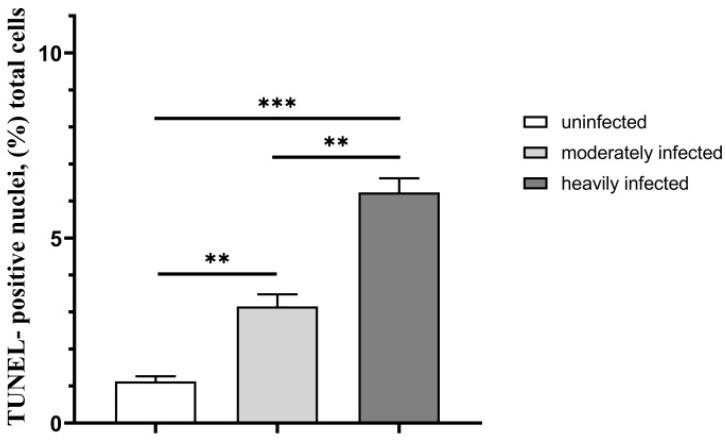
Quantitative assessment of TUNEL-positive cells in VNC of crabs, *Hemigrapsus sanguineus*, uninfected, moderately infected, and heavily infected by *Cercaria fluviocinguli*. The histogram shows the percentage of TUNEL-positive nuclei relative to the total number of cells. Data analysis was performed in GraphPad Prism 7 using the one-way analysis of variance (ANOVA) followed by Tukey’s multiple comparison test. Data are presented as mean ± SEM (*n* = 6); ** *p* < 0.01; *** *p* < 0.001.

A comparative quantitative analysis of TUNEL-positive cells of the VNC in crabs with different degrees of infection showed that the percentage of TUNEL-positive host cells in the uninfected crabs was low (1.13 ± 0.13% of the total number of host cells) (Figure 6). In the moderately infected crabs, *C. fluviocinguli* induced an insignificant increase in the percentage of TUNEL-positive host nuclei to 3.15 ± 0.33% (ANOVA, *p* < 0.01) (Figure 6). As shown in Figure 6, the percentage of TUNEL-positive nuclei was also increased in the VNC of the crabs with high levels of infection, reaching 6.23 ± 0.38% (Figure 6).

## 3. Discussion

In this study, we investigated the effects of infection caused by metacercariae of the trematode *Cercaria fluviocinguli* on the 5-HT-like immunoreactivity, inflammation, and pathological processes in the nervous system of the shore crab *Hemigrapsus sanguineus*.

As our results showed, in the VNC of infected crabs, metacercariae were present in the neuropil but were aggregated primarily in the nerves extending from the TG and innervating the legs. The mixed nerves of the TG included both sensory and motor axons [47]. Intensive colonization of these areas by metacercariae of *C. fluviocinguli* caused mechanical distortion of the host’s nerve fibers and altered the normal architecture of the leg nerves. As is known, damage to axons of sensory neurons may render crabs insensitive to external stimuli, whereas damage to motor neuron axons may impair transmission to the neuromuscular junctions. Thus, it is likely that the metacercariae in nerves could act as physical roadblocks and affect the transmission of nerve impulses in this processing center that integrates sensory information from the appendages, produces outputs in motor nerves to the body and limb muscles, and controls locomotion. Our data are consistent with results of a study by Sparks and Hibbits [29] who found encysted metacercariae in the TG nerves in crabs, *Cancer magister*. As the authors showed, the metacercariae, which presumably belonged to the family Microphallidae, affected the transmission of nerve impulses and caused ataxic behavior in crabs *C. magister* infected by multiple cysts that were found in the nerves of the TG.

Larvae of parasitic helminths can also affect neuromodulation [1,22]. In crustaceans, the VNC is a vital center whose neurons express a variety of neurotransmitters and hormones, one of which is serotonin (5-HT), potentially involved in parasitic manipulation of invertebrates (see review [11,18,20]). It may act as a neurotransmitter/neuromodulator or a neurohormone and plays crucial roles in numerous physiological processes (such as food intake, posture, cardiovascular function, modulation of muscle contraction and sensorimotor pathways, neural plasticity, aggression, locomotion, and walking) that regulate physiological and metabolic functions, energy homeostasis, and behavior in both vertebrates and invertebrates [48,49,50]. In crustaceans, 5-HT has been shown to modify synaptic transmission and endogenous properties of VNC neurons and also facilitate transmitter release at the neuromuscular junction [51,52].

Our immunohistochemical examination showed that in *H. sanguineus*, as in other crustacean species [53,54], 5-HT-synthesizing neurons are present in all regions of the VNC. In the uninfected crabs, we found intense 5-HT-immunopositive staining of extensive plexuses in the neuropils of the TG and SEG. However, in the VNC of the infected crabs *H. sanguineus*, 5-HT labeling in small varicose fibers in the neuropils of the TG and nerves decreased to almost complete absence of all 5-HT-lir neurons and nerve fibers in the TG and SEG of the VNC in the heavily infected individuals, which may indicate an effect of metacercariae on serotonergic signaling. Studies that investigated the response of crustacean hosts to a parasitic infection showed that 5-HT levels in the nerve ganglia and hemolymph of infected crabs are changed [21,23,30,51], being dependent on the parasite species, its localization in the host body, and the stage and extent of infection. These differences may reflect diverse molecular strategies employed by different parasite species or a differential response exhibited by different host species. Since the 5-HT system has a variety of roles in crustaceans [48,49,50,51,52], a disruption in serotonergic signaling may affect behavioral responses, from altered locomotor activity [52] to variations in aggression levels [55,56]. It is known that representatives of the genus *Maritrema* can decrease 5-HT levels in their host shore crabs, thus causing them to be less responsive to approaching danger [22].

A noteworthy feature of the interaction between *C. fluviocinguli* metacercariae and *H. sanguineus* was the high 5-HT-lir levels in tissues of the parasite. In our study, some of the metacercariae were found encysted in the VNC, nerves, connective tissue, and muscles, while others were not enclosed in a cyst wall, which indicated a likelihood of direct parasite–host interaction. Some cerebral microphallids are known to produce 5-HT and regulate its level in the decapod nervous tissue [23]. 5-HT is an excitatory neurotransmitter in parasitic flatworms—cestodes, trematodes, and monogeneans—that causes contraction of their muscles [57,58]. Trematodes are characterized by intense 5-HT-lir in the muscles of the oral and abdominal suckers, where 5-HT is critical for attachment and movement within the host [59]. For example, it has been experimentally shown that exposure to 5-HT significantly increases motility of newly excysted juveniles of *F. hepatica* [60]. Although trematodes can synthesize serotonin, they are also able to actively absorb it from host tissues through a specialized transport system [61,62]. The 5-HT system, in particular 5-HT7-type receptors, mediates motor control, which allows larvae to respond to the host environment and potentially manipulate host behavior [59,60].

Our results showed pathological changes in the neuropils of the VNC in the heavily infected crabs *H. sanguineus*, which might be a consequence of mechanical occlusion and deformations of nerves and chronic neuroinflammation caused by *C. fluviocinguli* metacercariae. Pathological tissue damage from metacercariae was also reported for fish infected by *Clinostomum piscidium* [63]. Previously, evidence was presented that the toxic effects of parasites’ excretory-secretory (ES) products such as enzymes, allergens, and immunomodulators can lead to tissue damage, oxidative stress, disrupt immune responses, or cause inflammation [64]. For example, a cysteine protease that can break down host proteins was identified in metacercariae of clinostomids [65]. This enzyme is essential for immature flukes as it facilitates their movement and development in host tissues.

**Figure 7:**
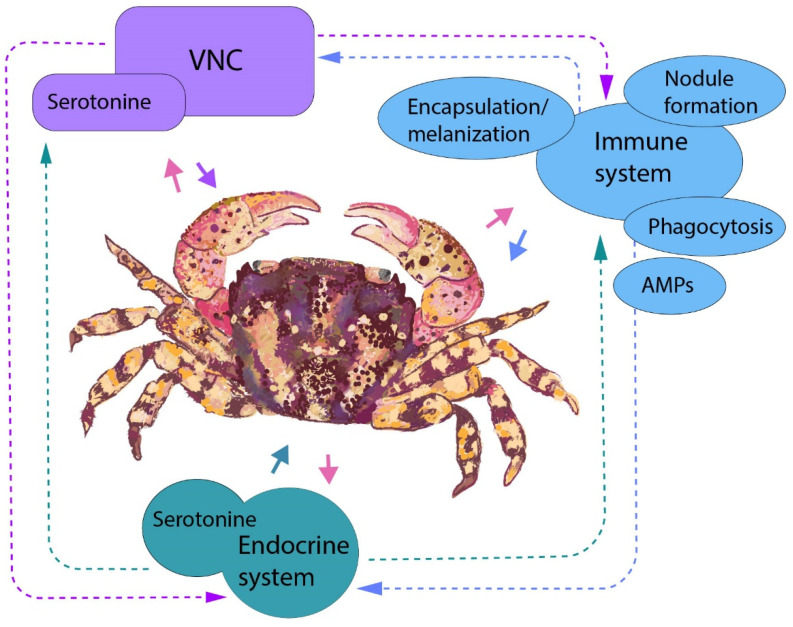
A hypothetical model for possible involvement of the nervous, endocrine, and immune systems of *Hemigrapsus sanguineus* in the response to metacercariae of *Cercaria fluviocinguli* based on observations in the present study and previously published data [21,31,51,67,68,69,70,71,72,73,74,77,79,80,82]. The metacercaria induces the 5-HT production in the VNC; 5-HT stimulates the release of neurohormones of the endocrine system into the hemolymph; neurohormones and 5-HT act on hemocytes, stimulating the immune system; the cooperative action of the cellular-mediated (phagocytosis, encapsulation, and nodule formation) and humoral-mediated responses (prophenoloxidase-activating system, antimicrobial peptides, etc.); hemocytes produce signaling molecules which act on the nervous and endocrine systems; immune responses are modulated by neuroendocrine signals. Letter designations: VNC, ventral nerve cord; AMPs, antimicrobial peptides.

According to the literature surveyed, parasitic infections, in addition to the nervous and endocrine systems, cause activation of the immune system in the host body, which triggers a complex defense mechanism involving multiple immune cells along with synthesis of various signaling molecules [66,67,68,69,70,71,72] (Figure 7). In decapod crustaceans, both cellular and humoral immunities are mediated by hemocytes (hyaline cells, semi-granular cells, and granular cells) and free-cell components in the hemolymph [73,74,75].

In heavily infected *H. sanguineus*, the immune response is accompanied by the formation of hemocytic aggregates and melanized foci corresponding to granulomas [76]. Such encapsulating granulomas, indicating chronic inflammatory lesions, were previously recorded from claw muscles, gills, the heart, and the gut of crustaceans as an immune response to parasitic infections [76,77,78]. In our study, granulomas in the infected *H. sanguineus* occurred exclusively in the VNC. The encapsulation caused a multilayered, overlapping sheath of hemocytes to form around the parasite that finally was eliminated within the capsule [73]. For many crustaceans, the encapsulation response to trematode infection indicates a strong host immune reaction, which results in successful walling off or killing of parasites [30,77] and is mediated either by the prophenoloxidase (proPO) cascade leading to melanization or by the production of free radicals and antimicrobial peptides (AMPs) [71,72,73,77,79]. In arthropods, the melanization could be an effective immune strategy to fight infection [71,72]. However, it is known that chronic granulomatous inflammation, characterized by excessive or long-lasting production of cytotoxic substances during melanization that have enzymatic and DNA-damaging properties and contribute to the destruction of parasites, can also eventually lead to permanent damage of host tissue and cell death [72,79,80].

In the present study, the apoptotic signal began to appear in TG neurons only in the moderately infected crabs. In the heavily infected crabs, the percentage of apoptotic cells, including neurons in the VNC, increased. The correlation between the number of dead neurons in the TG of the heavily infected crabs and the numerous hemocytic aggregates and melanized foci suggests that the cell death we observed could be associated with a high level of melanization. Furthermore, in the heavily infected crabs, different types of TUNEL-positive hemocytes were detected in granulomas of the VNC. However, further study is required to clarify which hemocyte types undergo apoptosis in granulomas. There is ample evidence that apoptosis of defense system cells can be beneficial to the host as it provides removal of excess cells and thereby avoidance of the detrimental effect of excessive inflammatory reactions in tissues [81,82]. In crustaceans, apoptotic cells are removed through hemocyte-mediated phagocytosis [82], a key step in the degradation of apoptotic, damaged, or unwanted cells. In crustaceans, as animals that lack an adaptive immune system, apoptosis, together with phagocytosis and encapsulation, can limit the spread of parasites [77,82].

## 4. Materials and Methods

### 4.1. Animals

Adult male shore crabs, *Hemigrapsus sanguineus*, measured 40–45 mm in carapace width, were captured in Peter the Great Bay (Sea of Japan). The crabs were placed in plastic tanks (140 L) with constantly aerated natural seawater and kept under normoxic conditions: a dissolved oxygen concentration of 6.5 ± 0.2 mg/L, a water temperature of 18 ± 0.5 °C, a salinity of 32‰, and a natural photoperiod of 12h/12h light/dark. The crabs were fed walleye pollock meat once a day. After a 1-week acclimation period, all the crabs were anesthetized by submersion in ice-seawater slurry. In each of them, the carapace was removed, and the hepatopancreas, gills, and muscle tissues (legs and cephalothorax) were examined for parasites. The internal organs and gills were pressed between two Petri dishes and examined under a stereomicroscope for metacercarial cysts. Metacercariae were isolated from muscle tissues by a previously described technique [83]: the tissues from the legs and cephalothorax were digested in a solution of artificial gastric juice (0.1% pepsin in 0.7% HCl) at 37–45 °C for 3–10 h in a shaking incubator. Then the homogenate was filtered, washed repeatedly with saline, and examined for metacercariae in a Petri dish under a stereomicroscope. As the cysts were semi-transparent, the parasites could be seen inside and recovered manually by pricking the cyst wall with a fine needle.

For histological examination, the brain with the eyestalks and the ventral nerve cord (VNC) with surrounding tissues and nerves from each crab were fixed with 4% paraformaldehyde solution (PFA; Sigma-Aldrich, St. Louis, MI, USA) in phosphate-buffered saline (PBS) at 4°C for 4 h. After several washes with PBS, the specimens were frozen and cut into 20 μm sections on a Cryo-Star HM560 MV cryostat (Thermo Fisher Scientific, Waltham, MA, USA) which were then mounted on glass slides. Frozen sections were examined for parasites under a light microscope. The sections were then used for immunohistological studies. Only crabs infected by a single trematode species, *Cercaria fluviocinguli*, were used in the study. This trematode was identified and described earlier elsewhere [84,85]. Crabs affected by mixed infections were discarded.

A total of 35 crabs containing *C. fluviocinguli* metacercariae and 10 control (uninfected) crabs were selected for the study. The crabs were categorized [86,87] into infected and uninfected groups based on the total number of metacercariae in their bodies counted after full dissection: uninfected (0 metacercariae, 10 crabs); moderately infected (<30 metacercariae, 20 crabs); and heavily infected (>30 metacercariae, 15 crabs).

### 4.2. Immunohistochemistry

For immunohistochemical staining, the cryostat sections were rinsed with PBT (PBS + 0.1% Triton-X100). The specimens were then blocked with 1% bovine serum albumin (BSA; Millipore, Burlington, MA, USA) in PBT for 12 h and incubated with a primary antibody diluted in the blocking solution at 4 °C overnight. To visualize 5-HT, a polyclonal rabbit antibody was used (1:1000; ImmunoStar Inc., Hudson, WI, USA, #20080). According to the manufacturer’s instructions, staining with this antibody was completely eliminated upon pretreatment with 25 mg of the 5-HT–BSA conjugate per 1 mL of diluted antibody. The overnight preincubation of the antibody with 10 mg/mL of the conjugate (20080, ImmunoStar Inc., Hudson, WI, USA) at 4 °C completely eliminated the 5-HT immunolabeling in control tissues. Furthermore, the overnight preadsorption of the diluted antibody with 10 mg/mL BSA at 4 °C did not affect this staining (i.e., this antibody recognized 5-HT alone, not BSA). This anti-5-HT antibody was previously used to detect 5-HT in the crustacean CNS [53,88].

To visualize the neuropil structure of the VNC, sections were incubated with mouse monoclonal anti-synapsin antibody (1:1000; clone 3C11 (anti SYNORF1), DSHB; Developmental Studies Hybridoma Bank, Iowa City, IA, USA), which targets a presynaptic marker (SYNORF1 or antibody 3C11). A previous study showed that this antibody detects an epitope widely conserved in the nervous system of arthropods, including crustaceans [89,90,91].

After the incubation with the primary antibody, the samples were washed thrice in PBT and incubated with 488-, 555-, or 647-Alexa Fluor conjugated donkey secondary antibodies (1:1000; Invitrogen, Thermo Fisher Scientific, Waltham, MA, USA) at 4 °C for 2 h. The sections were then rinsed thrice in PBS for 10 min each time, stained with the DAPI nuclei stain (1 μg/mL Sigma-Aldrich; Millipore, Burlington, MA, USA) in PBS at 22 °C for 2 h, rinsed with PBS, and embedded in glycerol (Sigma-Aldrich; Millipore, Burlington, MA, USA). In additional control experiments for possible nonspecific binding to the secondary antiserum, the primary antisera were omitted (5-HT and anti-synapsin antibody), replaced with blocking solution, and then followed by the labeling protocol as above. In these control experiments, staining was absent (Supplementary Figure S1A–F).

### 4.3. Terminal Deoxynucleotidyl Transferase dUTP Nick End Labeling (TUNEL) Assay

The TUNEL assay was carried out to quantify apoptotic nuclei in the VNC in infected and uninfected crabs. Staining was performed on sections attached to glass slides. The sections were washed and treated with a proteinase K working solution at 37 °C for 22 min. Then the sections were processed according to the protocols for using the TUNEL Andy Fluor 594 Apoptosis Detection Kit (ABP Biosciences, no. A050). The TdT reaction was performed at 37 °C in a humidified chamber for 1 h. Slides treated with DNase I (Qiagen Incorporated, Valencia, CA, USA) served as positive controls. Slides treated without TdT served as negative controls (Supplementary Figure S2A–C). Finally, the cells were stained with DAPI. The fluorescence signal was examined, and photographs were taken through a Zeiss LSM 700 confocal microscope. All experiments were set up in triplicate. Images were taken using the Zeiss Axio Vision 3.1 software (Carl Zeiss Jena GmbH, Jena, Germany).

### 4.4. Microscopy and Imaging

The sections stained with fluorescent dyes were imaged on a Zeiss LSM 710 laser scanning confocal microscope at the Far Eastern Center of Electron Microscopy, A.V. Zhirmunsky National Scientific Center of Marine Biology, Far Eastern Branch, Russian Academy of Sciences, Vladivostok, Russia. All images were examined, processed, and analyzed using the ImageJ 1.53 software (National Institutes of Health, Bethesda, MD, USA) for three-dimensional visualization and analysis of the confocal stacks. Each section was sequentially scanned for each fluorophore, then separate and overlaid images (of all three channels) were taken and subsequently converted into projected images using subsets of z-stacks. The converted images were saved in the TIFF format and transferred to the Photoshop CS6 software (Adobe, San Jose, CA, USA), where their contrast and brightness were adjusted for optimal clarity. The presented figures show the projections of maximum immunoreactivity.

### 4.5. Quantification and Statistical Analysis

Images of the crab VNC were taken for the control and infected groups using identical scan settings on a Zeiss LSM 780 confocal microscope. For quantification of TUNEL-positive nuclei, images were taken at a 20× magnification. The total cell number (using a nuclear counterstain, DAPI) and the number of TUNEL-positive cells were counted. TUNEL- and DAPI-positive nuclei were measured in three crabs from each of the control, moderately infected, and heavily infected groups. In each of the three crabs from these groups, six VNC sections were analyzed.

TUNEL-positive nuclei were counted using the ImageJ cell counter and were expressed as a percentage of the total number of DAPI-positive nuclei. All data were analyzed and figures composed using the Prism 7 software (GraphPad, San Diego, CA, USA). The TUNEL-positive cell counts from both infected and control crabs were analyzed by one-way analysis of variance (ANOVA) with Tukey’s multiple comparison test. The results are presented as mean ± standard error of the mean for each VNC. Differences were considered significant at *p* < 0.01 and *n* = 6.

## 5. Conclusions

Growing evidence indicates a complex interplay between the nervous, endocrine, and immune systems in response to trematode infections. As shown in this study, the effects of metacercariae of the trematode *C. fluviocinguli*, colonizing specific areas of VNC in shore crabs, include a decrease in serotonergic immunoreactivity in neurons and neuropils of the VNC, damage to nerve fibers and neurons of the VNC, inflammatory reactions, accumulation of granulomas, apoptosis, and impaired conduction in the mixed nerves of the thoracic ganglia controlling locomotion.

The data obtained provide a framework for understanding neuroendocrine and immune processes in crustaceans infected by trematodes. It highlights the need for further investigations into host–parasite interaction mechanisms under controlled experimental conditions in order to clarify the actual effects of encapsulated and non-encapsulated metacercariae on host behavior and their role in transmission to the next host.

## Data Availability

The original contributions presented in this study are included in the article. Further inquiries can be directed to the corresponding author.

## Supplementary Materials

The following supporting information can be downloaded at: https://www.mdpi.com/article/10.3390/ijms27135993/s1.
