# Supplementary material for: Parasite-Induced Changes in the Nervous System of the Shore Crab Hemigrapsus sanguineus
Source: Int J Mol Sci. 2026 Jul 3;27(13):5993. doi: 10.3390/ijms27135993 (PMC13361825; doi:10.3390/ijms27135993)
Supplement: Supplementary file 1 [file ijms-27-05993-s001.zip › ijms-4248893-supplementary.pdf]

Supplementary Material

Parasite-Induced Changes in the Nervous Systems of the Shore Crab *Hemigrapsus sanguineus*

Elena Kotsyuba and Vyacheslav Dyachuk *

A.V. Zhirmunsky National Scientific Center of Marine Biology, Far Eastern Branch, Russian Academy of Sciences, Vladivostok 690041, Russia

* Correspondence: slavad83@gmail.com

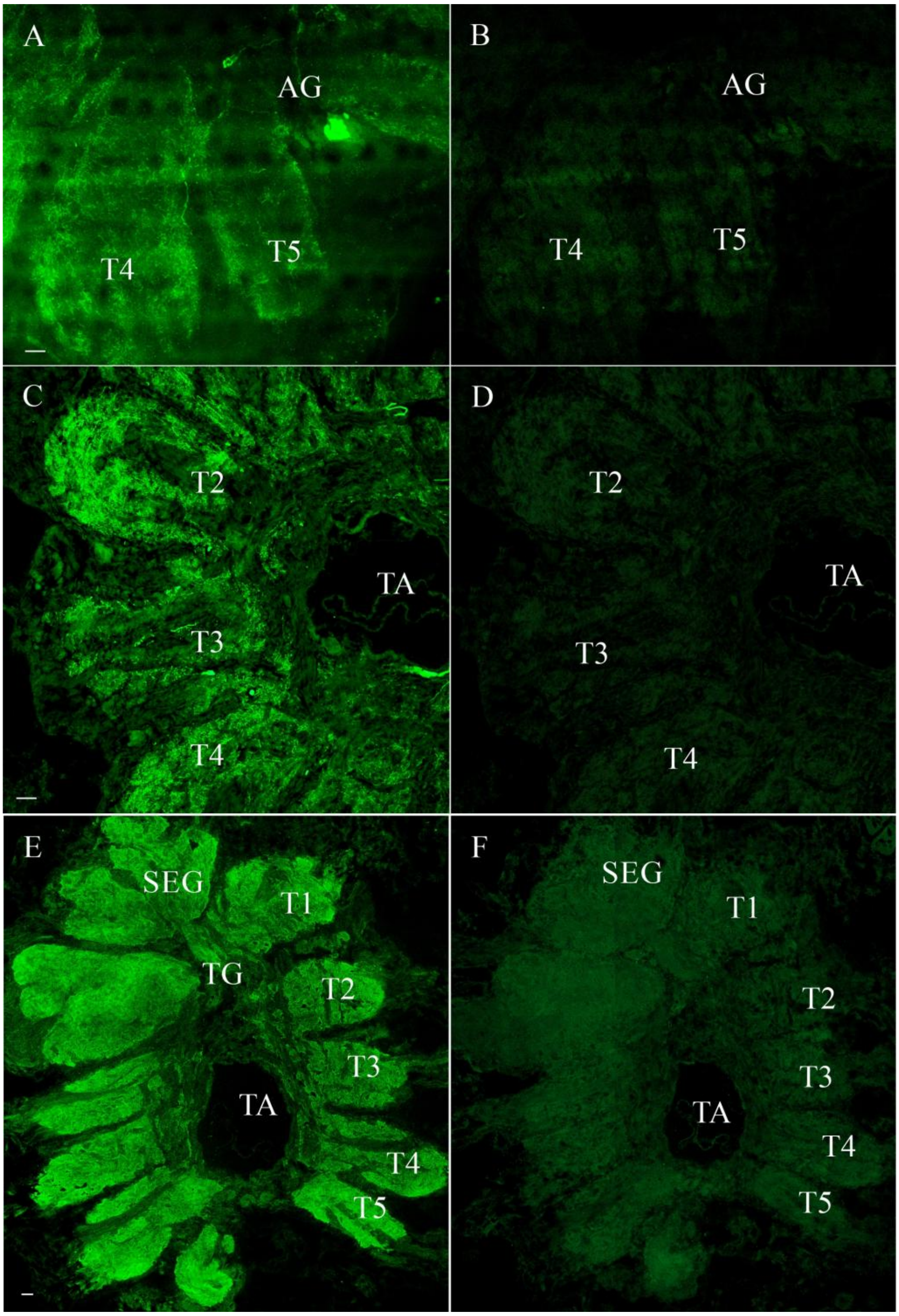

Supplementary Figure S1

Immunofluorescence detection of 5-HT-immunoreactivity (5-HT-lir) (A-D) and synapsin -immunoreactivity (syn-lir) (E-F) on serial slices of the VNC. (A) A cross section showing intense 5-HT-lir in large neuron of the AG and neuropils (T4-T5) of the TG. (B) A negative control micrograph showing no staining of 5-HT-lir in large neuron of the AG and neuropils (T4-T5). (C) A cross section showing intense 5-HT-lir in in fine varicose fibers of the neuropils (T2-T4) of the TG. (D) A negative control showing no staining of 5-HT-lir in the neuropils of the TG. (E) synapsin -immunoreactivity (syn-lir) in the neuropils of the VNC. (F) A negative control micrograph showing no staining of syn-lir in the neuropils of the VNC. Letter designations: SEG, subesophageal ganglion; TG, thoracic ganglion; AG, abdominal ganglion; TA, thoracic artery; T1–T5, neuropils of TG; Color designations: green, 5-HT and synapsin. Scale bars: 100 μm.

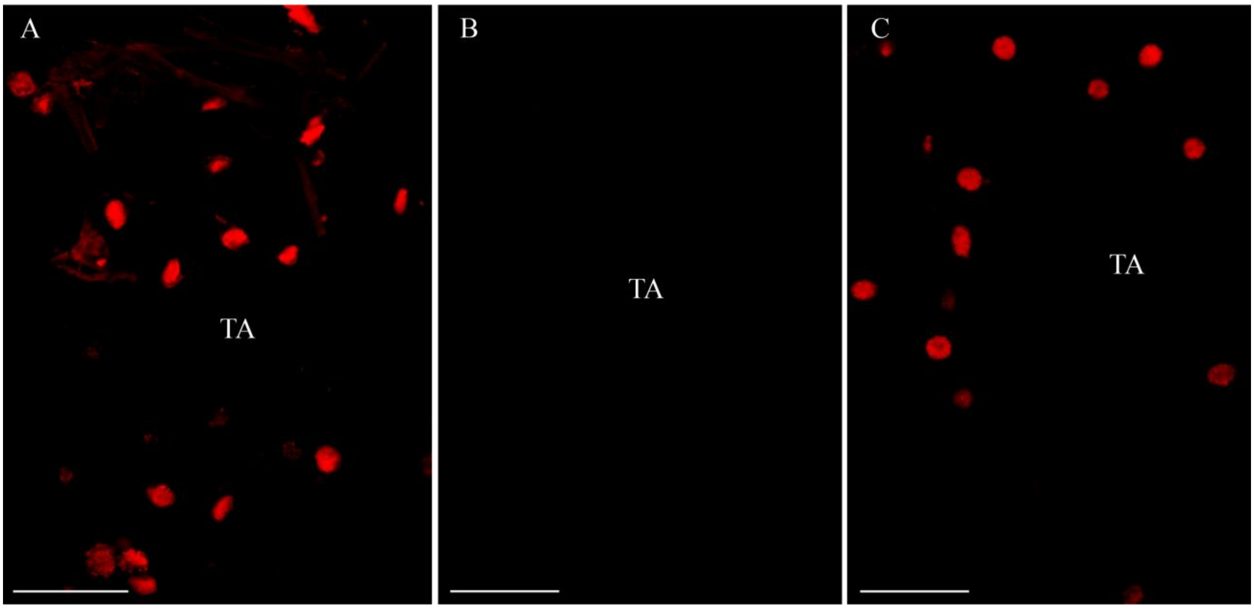

Supplementary Figure S2.
TUNEL assay analyze in thoracic artery. (A) Section treated with DNase I. (B). Negative control without TdT enzyme. (C) Nuclei stained with the TUNEL assay. Scale bars: 25 μm.
